# Additional Postoperative Radiotherapy Prolonged the Survival of Patients with I-IIA Small Cell Lung Cancer: Analysis of the SEER Database

**DOI:** 10.1155/2022/6280538

**Published:** 2022-06-18

**Authors:** Jiali Li, Zihang Zeng, Zhengrong Huang, Yan Gong, Conghua Xie

**Affiliations:** ^1^Department of Radiation and Medical Oncology, Zhongnan Hospital of Wuhan University, Wuhan, China; ^2^Department of Biological Repositories, Zhongnan Hospital of Wuhan University, Wuhan, China; ^3^Tumor Precision Diagnosis and Treatment Technology and Translational Medicine, Hubei Engineering Research Center, Zhongnan Hospital of Wuhan University, Wuhan, China; ^4^Hubei Key Laboratory of Tumor Biological Behaviors, Zhongnan Hospital of Wuhan University, Wuhan, China; ^5^Hubei Cancer Clinical Study Center, Zhongnan Hospital of Wuhan University, Wuhan, China

## Abstract

**Purpose:**

Complete resection and adjuvant chemotherapy are recommended as the standard strategy for patients with stage I-IIA small cell lung cancer (SCLC). However, the role of additional postoperative radiotherapy (PORT) in treatment remains controversial.

**Methods:**

Patients with stage I-IIA SCLC undergoing surgery and adjuvant chemotherapy were extracted from the Surveillance, Epidemiology, and End Results database. Stage I-IIA, defined as T1-2N0M0, was recalculated according to the 8th AJCC TNM staging system. Propensity score matching (PSM) was conducted to identify the therapeutic impact of PORT. Univariate Cox hazards regression and least absolute shrinkage and selection operator regression were utilized for primary screening of prognostic variables for I-IIA SCLC disease. A nomogram to predict overall survival (OS) was constructed based on the multivariate Cox proportional hazards model, evaluated with area under the curve, calibration curve, and decision curve analysis, and validated with bootstrap resampling.

**Results:**

Our results demonstrated that compared with no PORT, PORT significantly prolonged the median OS (8.58 vs. 5.17 years, HR = 0.61 [0.39–0.96], *P* = 0.032) and median cancer-specific survival (11.33 vs. 8.08, HR = 0.47 [0.27–0.82], *P* = 0.0086) after PSM. The 5-year OS rate was 61.56% vs. 46.60%. Five variables including age at diagnosis, gender, T stage, surgical type, and PORT were elucidated to impact on prognosis and included in a nomogram to predict 3-/5-/10-year OS probability. The area under the curve values were 0.72, 0.71, and 0.81, respectively. The nomogram also exhibited satisfactory accuracy and clinical usefulness.

**Conclusion:**

PORT was verified to improve the OS of patients with T1-2N0M0 SCLC after surgery and chemotherapy. A prognostic nomogram was developed and validated for OS prediction for these patients.

## 1. Introduction

Small cell lung cancer (SCLC) accounts for approximately 15% of lung cancer around the world [1]. With the gradual acceptance of the American Joint Committee on Cancer (AJCC) staging system and intensive research of SCLC, the role of surgical intervention in early patients has been gradually elucidated [2]. Surgical resection and adjuvant chemotherapy is the preferred strategy for patients with stage I-IIA (T1-2N0M0) SCLC, improving 5-year OS survival to 48% compared with the nonsurgical group [1, 3]. Platinum-based sequential chemotherapy is especially emphasized and proved to benefit postoperative SCLC patients at early stages [4], while postoperative radiotherapy (PORT) in stage I-IIA patients is implied to be undefined.

In 2016, a retrospective research derived from the National Cancer Database, enrolling 477 clinical cT1-2aN0M0 SCLC patients with R0 surgical resection, investigated the impact of PORT in the treatment paradigm in stage I SCLC (based on 7^th^ edition of AJCC stage) patients. Results indicated that PORT in combination with chemotherapy demonstrated no significance on 5-year overall survival (OS) in patients at pathologically confirmed stage T1-2aN0M0 versus those with adjuvant chemotherapy alone (*P*=0.89, 52% vs. 53%) [5].

Another study that included 3,017 limited stage SCLC cases with negative margins revealed the heterogeneous effects of PORT on OS based on the NCBD [6]. When stratified by pathological node, PORT was demonstrated to impose an inferior impact on pathological N0 patients, of which over 90% was diagnosed as T1-2, decreasing 5-year OS to 39% versus 43% in the surgery group.

A consistent conclusion has not been drawn on the roles of PORT in stage I-IIA SCLC patients constrained by rarity of SCLC and heterogeneity between groups among published retrospective research studies. With advanced diagnostic technologies and accumulating SCLC cases receiving operative intervention, it is possible to clarify the effects of PORT on the survival of T1-2N0M0 SCLC patients. We tried to solve the problem using propensity scoring matching (PSM) analysis to balance the covariates and construct a prognostic nomogram based on the Surveillance, Epidemiology, and End Results (SEER) database [7]. Our studies provided more evidence on the clinical decision-making process of curative measure selection for stage I-IIA SCLC patients.

## 2. Material and Methods

### 2.1. Data Collection

SEER is a population-based clinical oncology repository maintained by National Cancer Institute. It recorded information about incidence, mortality, and morbidity of confirmed frequent malignancies, updating itself on April by year. In this retrospective study, data of SCLC patients were retrieved from the SEER database by SEER^*∗*^Stat software (version 8.3.8), and therapy fields were accessed with a custom warrant (reference number 21330-Nov 2019) following the instruction of the recommended procedure [8].

Inclusion criteria were as follows: (1) age ≥18 years; (2) pathologically confirmed SCLC (ICD-O-3 histology was listed as 8002/3), small cell carcinoma, NOS (8041/3); oat cell carcinoma (8042/3), small cell carcinoma, fusiform cell (8043/3); and small cell carcinoma, intermediate cell (8044/3); (3) diagnosed as the first primary SCLC; (4) diagnosed as I-IIA SCLC based on the 8th edition of the AJCC Cancer Staging Manual (T1-2N0M0); (5) received primary site surgery with concrete operative type; (6) received chemotherapy as part of therapy strategy; and (7) with complete record of radiotherapy and active follow-up information. Patients with only autopsy/death certificates and those with intraoperative and/or preoperative radiotherapy were excluded from this study.

Variables extracted from the SEER database included age at diagnosis, gender, race recode, year of diagnosis, derived AJCC T stage, derived AJCC Stage Group, RX Summ-Surg Prim Site, chemotherapy recode, radiation sequence with surgery, tumor size summary (2016+), CS tumor size (2004–2015), sequence number, survival months, vital status recode, and SEER cause-specific death classification.

Of note, patients with primary site surgery and chemotherapy with or without PORT were enrolled in this study, while those with intraoperative and/or preoperative radiotherapy and/or other combinations were excluded. During preprocessing phase, AJCC T stages were recalculated based on tumor size, and AJCC stages were calibrated according to the AJCC 8^th^ edition tumor-node-metastasis (TNM) staging system. Specifically, SCLC patients with primary tumor size smaller than 5 cm, without positive lymph node or distant metastasis were included in the work. The TNM stages were clinically or pathologically diagnosed in the first course of their therapy. In addition, original information extracted from SEER^*∗*^Stat software is presented in Table S1.

SEER codes were used to stratify operative types: 12, 13, 15, 19–25 were defined as sublobar resection (including local tumor destruction and resection of less than one lobe); 30, 33, 45–48 were included in lobectomy or extended; and 40, 50, 51–56, 65–66, 70 represented for pneumonectomy or extended. All cases meeting the above criteria were diagnosed during 2004–2016.

### 2.2. Collinearity Diagnosis

Collinearity in statistics is defined as the explicability and correlation among variables, resulting in the instability of statistics. Spearman correlation analysis and variance inflation factor (VIF) were assessed to exclude the collinearity of covariates. Covariates with Spearman coefficients larger than 0.4 or VIF >4 were defined as dependent variables and excluded from downstream analyses. Categorical variables were transformed into dummy variables to get involved in the diagnostic process. Correlation coefficients between dummy variables of the same covariate were not applicable to the above principles.

### 2.3. Propensity Score Matching Analysis

PSM is an algorithm applied to eliminate the deviations in baseline characteristics between groups. Covariates screened out by collinearity diagnosis were involved in the pairing process. Caliper and ratio values were adjusted to exert a balance between the PORT and no-PORT groups.

### 2.4. Nomogram Construction, Estimation, and Validation

The least absolute shrinkage and selection operator (Lasso) regression and univariate Cox regression were performed to screen prognostic features for the nomogram. Covariates with minimal deviance were selected based on 10-fold cross-validation using glmnet package. The optimum variable combination was decided based on the minimal Akaike information criterion (AIC) value. To assess the time-to-event outcome, such as the 3/5/10-year OS probability, the multivariate Cox proportional hazards model was selected to construct the nomogram. The performance of the nomogram was evaluated from three aspects: discrimination, calibration, and clinical utility. The area under the receiver (AUC) operating characteristic curve and time-dependent AUC were calculated to appraise the discriminative ability of the established nomogram. A calibration plot was used to estimate the consistency between predicted and actual survival probability. Decision curve analysis (DCA) was applied to evaluate the clinical benefit of intervention at different threshold probabilities. Treat-all and treat-none strategies were used as comparisons. The area under the decision curve (AUDC) was reported to quantify the “net benefit treated” values, which denoted the subtraction between expected profit and expected loss for patients who were treated with PORT.

To verify the nomogram, resampling technique (*N* = 1,000) by bootstrapping was adopted for overall performance estimation. The AUC and its 95% confidence interval of the model for 3/5/10-year OS probability were obtained to measure the model's accuracy based on the 1,000 resampling copies.

### 2.5. Statistical Analysis

All the statistical processes were performed with R software (version 3.6.1). The Shapiro–Wilk test was performed to define normal distribution of quantitative variables. Proportional hazards assumption was verified with Cox.zph in survival package. Univariate Cox hazards ratios were calculated to primarily estimate the impact of a factor on prognosis. Survival curves were depicted based on the Kaplan–Meier plot, and differences in survival were distinguished with the log-rank test. The quantitative variables of normal distribution or nonsevere skewness distribution were differentiated using the *t*-test. The categorical clinical characteristics between groups were analyzed with the chi-square test. In all hypothesis tests, 2-sided *P* values less than 0.05 were regarded as statistically significant.

## 3. Results

### 3.1. Clinical Characteristics of SCLC Patients

**Figure 1:**
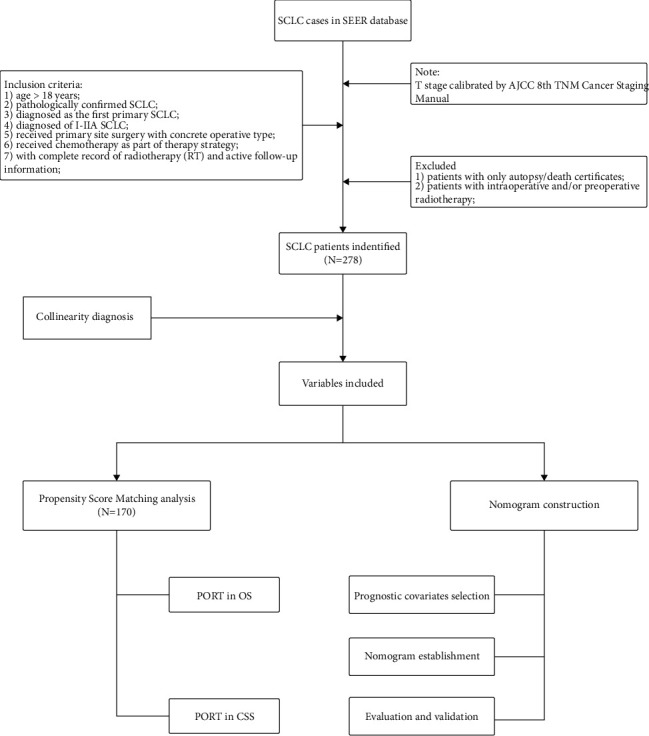
Workflow of this study. SEER: Surveillance, Epidemiology, and End Results; SCLC: small cell lung cancer; RT: radiotherapy; PORT: postoperative radiotherapy; OS: overall survival; CSS: cancer-specific survival.

A total of 278 SCLC patients diagnosed as T1-2N0M0 stage met the criteria and were included in this study (Figure 1). Median age at diagnosis was 66.0 years (interquartile range 59.0–71.0 years). An active median follow-up was 7 years with 95% confidence interval (CI) of 4.25–8.58. In total, 178 SCLC patients underwent primary site surgery and adjuvant chemotherapy, and 100 patients received additional PORT. No significant difference was observed between PORT and no-PORT patients in age, gender, race, year of diagnosis, specific stage, and tumor size (*P* > 0.05). The tumor sizes in both groups were around 2 cm (*P*=0.453), and sex was evenly distributed (*P*=1). Over 90% of the population was diagnosed during 2004–2015, and most of the patients were white (94.4% in PORT group vs. 97% in the other). Moreover, lobectomy or extended was the primary surgical type, although a smaller proportion (59%) was recorded in the PORT group compared with 69% in no-PORT patients (*P*=0.079). The raw information of the enrolled cohort is presented in Table S1, and the clinical characteristics and demographics of these SCLC patients are summarized in Table 1.

### 3.2. Collinearity Diagnosis and PSM Analysis

Spearman correlation analysis and VIF were performed sequentially to exclude the effects of collinearity covariates. The stage was discovered as the alias of T stage (*R* = −1), and tumor size was tightly associated with T stage (*R* > 0.4, Figure S1). In addition, 6 variables (age, gender, race, T stage, surgery type, and radiotherapy) were reserved for downstream analyses. In line with this, VIF values of these 6 variables were all <4, elucidating no collinearity between covariates (Table S2).

**Figure 2:**
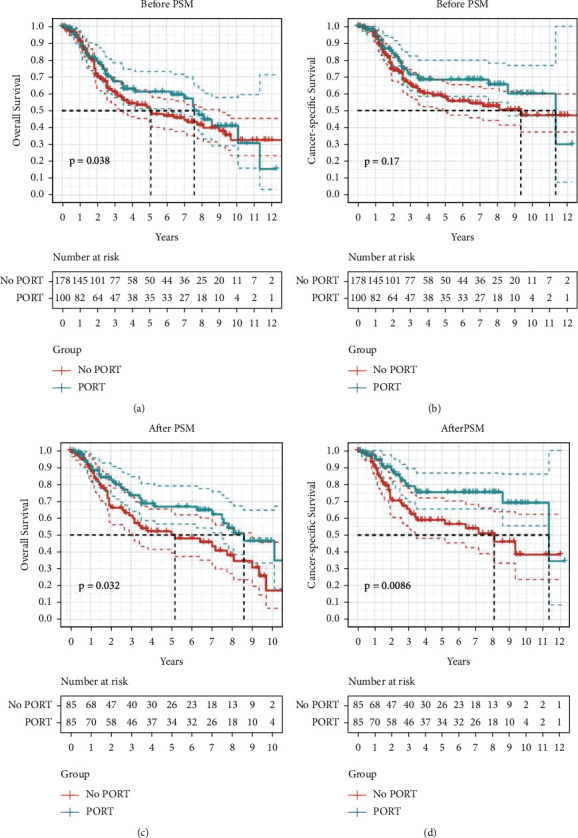
Survival curves for stage I-IIA SCLC patients with/without PORT before and after PSM. (a, b) Before PSM, additional PORT demonstrated no significance on OS (a) and CSS (b) for stage I-IIA SCLC patients compared with bimodality (surgery plus adjuvant chemotherapy, median OS: 7.58 vs. 5.08 years, HR = 0.84 [0.59, 1.22], *P*=0.38; median CSS: 11.33 vs. 9.33 years, HR = 0.73 [0.47, 1.11], *P*=0.17). (c, d) After PSM, additional PORT prolonged the OS (c) and CSS (d) for stage I-IIA SCLC patients (median OS: 8.58 vs. 5.17, HR = 0.61 [0.39, 0.96], *P*=0.032; median CSS: 11.33 vs. 8.08, HR = 0.47 [0.27–0.82], *P*=0.0086). PSM: propensity score matching; PORT: postoperative radiotherapy.

Before PSM, univariate Cox regression for all populations demonstrated that elder age and male were the adverse prognostic features for OS (both *P* < 0.05, Table 2). Lobectomy or extended surgery prolonged both OS and cancer-specific survival (CSS) compared with sublobar resection (OS: HR = 0.38, 95% CI = 0.26–0.55, *P*=0.00; CSS: HR = 0.34, 95% CI = 0.22–0.52, *P*=0.00; Table 2). On contrast, additional PORT exerted no significant impact on OS and CSS based on the Kaplan–Meier plots (*P* > 0.05, Figures 2(a) and 2(b)). To specifically observe the effect of PORT on prognosis, 1 : 1 PSM analysis was applied with a caliper of 0.05 to balance the bias between the PORT and no-PORT cohorts.

After PSM, clinical characteristics including age, gender, race, T stage, and surgery type were equally comparable (Table 1). A total of 170 patients were matched in the PORT and no-PORT groups. Further statistical analysis indicated that additional PORT was a favorably prognostic factor and prolonged the median OS and CSS (median OS: 8.58 vs. 5.17, HR = 0.61, 95% CI = 0.39–0.96, *P*=0.032; median CSS: 11.33 vs. 8.08, HR = 0.47, 95% CI = 0.27–0.82, *P*=0.0086; Figures 2(c) and 2(d)). Notably, the 3-, 5-, and 10-year OS rates in the PORT and no-PORT groups were 73.62% vs. 61.56%, 66.80% vs. 50.09%, and 46.60% vs. 17.10%, respectively. In consistent with this, the 3/5/10-year CSS rates in the above groups were 78.94% vs. 65.59%, 75.44% vs. 58.84%, and 69.10% vs. 38.45%, respectively. In the long term, additional PORT improved the prognosis of T1-2N0M0 SCLC patients with surgical resection and chemotherapy.

### 3.3. Covariate Selection and Nomogram Construction

To screen the prognostic factors in SCLC patients, univariate Cox analysis and Lasso regression were simultaneously conducted. Three variables (age, gender, and surgery type) were significantly associated with the OS according to univariate regression analysis (all *P* < 0.05, Table 2). Five variables (age, gender, T, surgery type, and radiotherapy) were filtered by Lasso with minimal deviance based on 10-fold cross-validation. The covariate combination screened by Lasso had a minimal AIC value compared with the results of univariate analysis and was reserved to further construct the prognostic nomogram for T1-2N0M0 SCLC.

**Figure 3:**
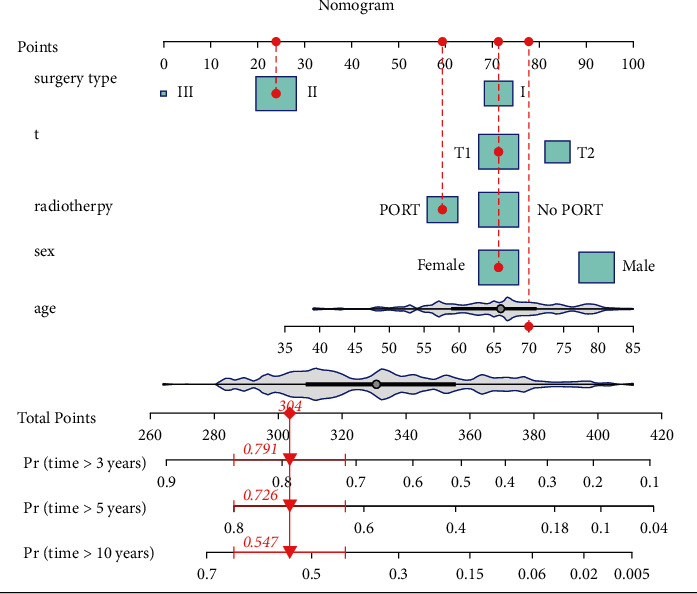
An example for utilization of proposed nomogram. A random patient was chosen from the population in this present study. She was a 70-year-old female with T1N0M0 SCLC who underwent lobectomy and chemotherapy. After PORT, the probability of her 10-year survival was 54.7% (0.95 CI [42.3%, 65.5%]). The violin plots exhibited the density distribution of the numeric variables age and total points. The box plots reflected the distribution of category variables. The red points on the “Points” line corresponded to score for each covariate. Total points equaled to the sum of scores for all covariates, which was 420 in our nomogram. The 3/5/10-year OS probability was predicted based on it.

The nomogram diagram was arranged according to the order of main effects in the model, with sequence of surgery type, T stage, radiotherapy, gender, and age. The total point was calculated as the sum of individual factors' scorings estimated by the nomogram, which was 420. The 3/5/10-year OS probability was predicted according to the total points.  Figure 3 is an example of a given patient's nomogram.

### 3.4. Performance Evaluation and Validation of Nomogram

**Figure 4:**
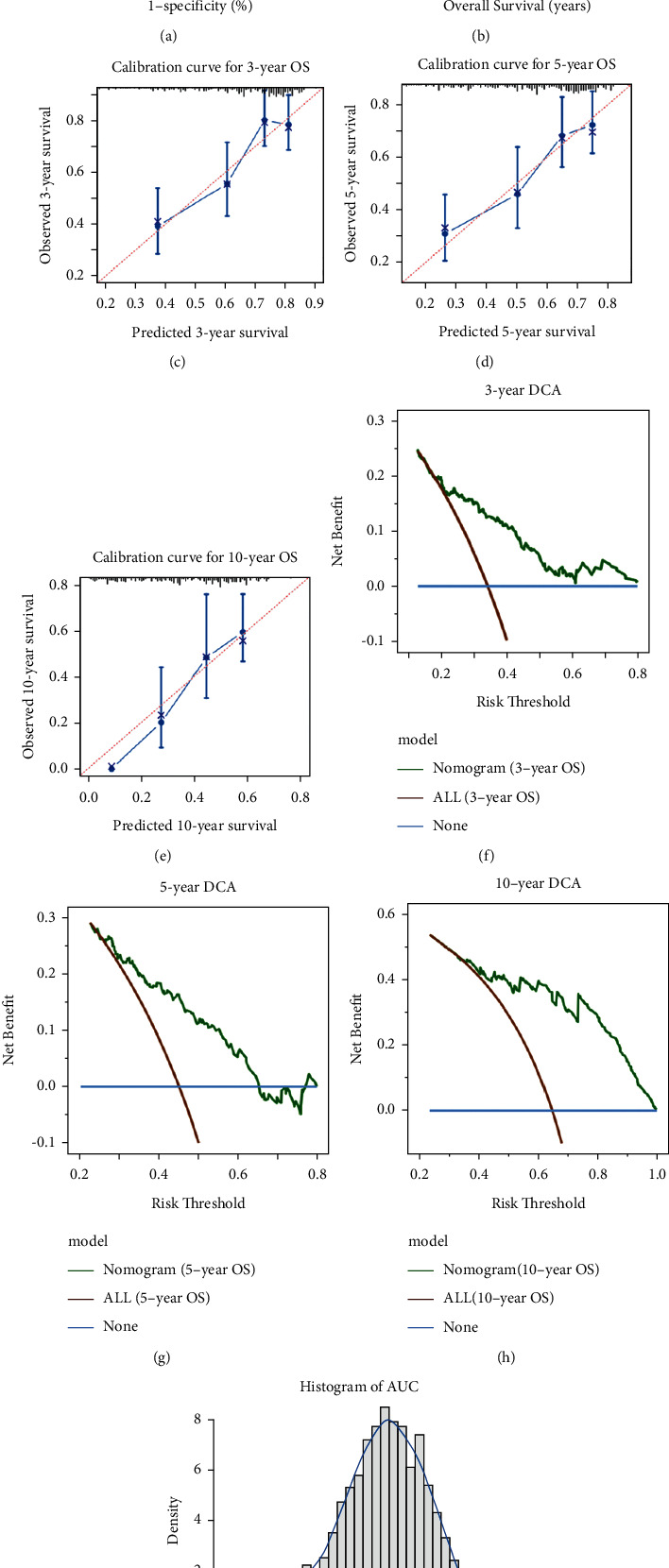
Evaluation and validation of performance of developed nomogram. (a) AUC of nomogram for the prediction of 10-year OS rate. (b) Time-dependent AUC of using the proposed nomogram to predict the OS within 10 years. The gray area represents the 0.95 CI of AUC at each time point. The dotted blue line reflects 0.65 AUC value. (c–e) Calibration curve for 3/5/10-year OS rate for patients with stage I-IIA SCLC. Calibration plots reflected the consistency between predicted OS probability by the nomogram and actual OS probability. (f–h) DCA plots of 3/5/10-year OS probability rate by the developed nomogram. Greater area among three curves accompanied with greater clinical practicability. (i) Frequency density diagram of AUC values using the nomogram for 10-year OS rate prediction based on the bootstrapping method (resample = 1,000). The CI of population parameter of AUC for 10-year survival prediction was evaluated as [0.7085, 0.9011]. ROC: receiver operating characteristic curve; AUC: area under the curve; OS: overall survival; DCA: decision curve analysis.

The proposed nomogram was estimated from discrimination, calibration, and clinical usefulness. The AUC of the nomogram for 3/5/10-year OS prediction was 0.72 (95% CI = 0.65–0.79), 0.71 (95% CI = 0.64–0.79), and 0.81 (95% CI = 0.73–0.90), respectively (Figure 4(a)). The time-dependent AUC was >0.65 for the prediction of OS rate within 10 years, demonstrating a comparably favorable discriminative efficacy (Figure 4(b)). In parallel, the calibration curves of 3/5/10-year OS probability by the nomogram were plotted, reflecting a high consistency between predicted and actual risks (Figures 4(c)–4(e)). Decision curve analysis was conducted to depict the clinical net benefits of OS with intervention under different threshold survival probabilities. The net benefit of the nomogram was higher than that of delivering PORT to all patients when the threshold probability was greater than 20% in 3-year survival, 26% in 5-year survival, and 34% in 10-year survival (Figures 4(f)–4(h)). The AUDC was 0.72 (95% CI = 0.65–0.79), 0.72 (95% CI = 0.64–0.79), and 0.82 (95% CI = 0.73–0.90), respectively.

To internally validate the discriminative ability and accuracy of our nomogram, the bootstrapping method (resample = 1,000) was performed to estimate the population parameter of AUC and calibrate event probability. The 95% CI of estimated parameter AUC for 3/5/10-year OS prediction were 0.64–0.78, 0.63–0.78, and 0.71–0.90, respectively, verifying the excellent discrimination of the nomogram.  Figure 4(i) exhibited the frequency density diagram of AUC values for 10-year OS prediction with bootstrapping.

## 4. Discussion

In the present study, PORT was demonstrated benefit of both the overall survial and cancer-specific survival of I-IIA SCLC patients with surgical resection and adjuvant chemotherapy after balancing baseline heterogeneity of the population by PSM. Moreover, we identified 5 prognostic variables including age, gender, T stage, surgical type, as well as PORT and proposed a nomogram to predict the OS probability using Lasso-COX regression. Following analyses demonstrated favorable discriminative capability, calibration capability, and clinical usefulness of our constructed nomogram. Bootstrap resampling was conducted for internal validation.

Based on the positive outcomes of previous clinical trials, the National Comprehensive Cancer Network (NCCN) recommended surgical intervention and postoperative chemotherapy (POCT) as the rationale for I-IIA SCLC patients [3]. However, the role of PORT in I-IIA SCLC patients remained controversial. A number of studies demonstrated heterogeneous conclusions on this subject, which suffered from biases to some degree [6, 9–11]. In 2010, a retrospective research collecting stage I SCLC from 1988 to 2004 reported that the 3- and 5-year OS for patients with lobectomy and RT (*N* = 205) were 64.9% and 57.1%, and 50.3% of those without RT (*N* = 38), concluding that PORT was not suggested for these patients, with *P*=0.9 [9]. However, the lack of chemotherapy records and reclassification of stages were discovered in this study. Along similar lines, another study from the National Cancer Database published in 2016 clarified that no significance was observed when contrasting the effects on 5-year survival of patients with T1-2aN0M0 SCLC in postoperative chemo-radiotherapy and POCT cohorts (53% vs. 52%, *N* = 360, *P*=0.89) [5]. At the same time, PORT was investigated to impose an inferior impact on pT1-2N0M0, decreasing 5-year OS from 43% to 39% compared with surgical resection, whereas clinical characteristics including chemotherapy and surgical type between groups were found to be heterogeneous (*P* < 0.05) [6]. By contrast, Jin et al. revealed in a stratified analysis that PORT, relative to surgery alone, exerted a protective effect on the OS of T1N0M0 SCLC patients (*P*=0.014) but exhibited no significance in T2N0M0 cases (*P*=0.633). However, chemotherapy information was not accessible in the study [10]. Taken together, due to the incomplete record or different stages between enrollment subjects, no agreement on the impact of additional PORT was achieved in I-IIA SCLC patients. In our study, both surgery type and chemotherapy records were fully accessed, and discrepancy of clinical characteristics between the PORT and no-PORT group were eliminated through PSM. Notably, tumor T stages were recalculated to suit for the 8^th^ edition AJCC staging system. Based on the above processes, we concluded that additional PORT significantly prolonged the median OS to 8.58 years (vs. 5.17, HR = 0.61, 95% CI = 0.39–0.96, *P*=0.032) and improved the 5-year OS probability to 61.56% (vs. 46.60%).

In addition to classifying the roles of PORT in stage I-IIA SCLC, prognostic characteristics were identified in the present study. Earlier age at diagnosis, female, T1 stage, lobectomy or extended surgical scope, and PORT administration were favorable factors for patients with primary site operation and POCT. These factors were logically sensible and consistent with previous studies [12–17]. In terms of operative type, a quantity of literature elucidated the priority of lobectomy compared with sublobar resection for stage I-II SCLC, which accorded with our study [13–15]. Particularly, a larger surgical scope as pneumonectomy was not accompanied with better survival for re-stage I-IIA SCLC according to the 8th edition TNM staging system [13]. This probably attributed to surgical damage to normal tissue and the impact on pulmonary function [18, 19]. Meanwhile, adjuvant therapy was another controversial factor [12, 14, 17]. Lin et al. implicated that trimodality led to better survival than surgery alone (adjusted HR, 0.543; 95% CI = 0.331–0.889) and bimodality (surgery plus POCT, adjusted HR, 0.641; 95% CI = 0.389–1.057) in T1N0M0 SCLC patients [14], suggesting the inclusion of PORT in first-course treatment. Other identified variables such as female, age, and earlier T stage were indicated as beneficial factors associated with improved OS and CSS of stage I SCLC in literature as well [12, 17].

During the process, both univariate Cox analysis and Lasso regression were conducted for significant variable screening for SCLC patients with stage I-IIA. Identified covariates by both methods were subjected to multivariable Cox hazard ratios to construct prognostic models. For comparison, the minimal Akaike information criterion (AIC) functioned as the evaluation criterion of goodness of fit. Of note, the introduction of L1 regulation to loss function enabled a better shrinkage and fitting capability. With a comparable smaller AIC, the Lasso-Cox model outperformed the combination of Cox regression. Actually, Lasso-Cox was adopted in extensive research to determine prognostic molecular features or clinical characteristics [20–22]. For instance, the group Lasso-Cox model was executed to predict patient prognosis and identify risk protein complexes in glioblastoma multiforme, ovarian cancer, and lung adenocarcinoma. In this work, we provided an example to verify the priority of this popularly used method based on statistic estimation standard.

Using the screened 5 covariates, a prognostic nomogram was proposed to estimate the 3/5/10-year OS probability of stage I-IIA SCLC. The AUC were, respectively, 0.72, 0.71, and 0.81, all higher than 0.7, indicating a reasonable prediction capability. The risk thresholds of net benefit for clinical intervention were also defined according to DCA. Furthermore, the bootstrapping method (resample = 1,000) was performed to validate the calibration and discriminative ability. To summarize, our model enrolled all stages that were suitable for surgery based on NCCN guidelines and was the first nomogram to predict OS for stage I-IIA SCLC patients. For comparison, the similar nomograms proposed by previous studies focused on patients with stage I SCLC, overlooking the IIA-stage patients. Further observations revealed these models targeted the CSS as the endpoint, lacked the calibration of T stage, and demonstrated less effective diagnostic performance (all, C-index <0.7) [17, 23]. In addition, our model outstood in the prediction of 10-year OS probability, indicating long-term benefit with this nomogram.

Although we tried to minimize the biases in this study, it had some limitations. For instance, certain information was not included or inaccessible in SEER, like the sequence of chemotherapy and surgery, the status of surgical margins, and performance scores of patients. In addition, considering an inadequate sample capacity, multicenter collaboration was needed to provide further external validation of impact of PORT and nomogram in T1-2N0M0 SCLC patients.

## 5. Conclusion

Our study demonstrated that additional PORT was suggested for patients with T1-2N0M0 SCLC as it prolonged the overall survival. A nomogram that incorporated age, gender, T stage, surgical type, and PORT was established and validated to achieve satisfactory prediction of 3/5/10-year OS probability from discriminative efficacy, concordance, and clinical usefulness.

## Figures and Tables

**Table 1:** Characteristics and demographics of SCLC patients.

	Before PSM					After PSM			
	Total	No PORT	PORT	*P* value		Total	No PORT	PORT	*P* value
	278	*N* = 178	*N* = 100			170	*N* = 85	*N* = 85	
Age	278	67.0 [59.0; 72.0]	65.5 [59.8; 70.0]	0.364	**Age**	170	65.0 (8.97)	65.5 (8.06)	0.699
*Gender*				1	**Gender**				1
Female	157	101 (56.7%)	56 (56.0%)		Female	95	48 (56.5%)	47 (55.3%)	
Male	121	77 (43.3%)	44 (44.0%)		Male	75	37 (43.5%)	38 (44.7%)	
**Race**				0.423	**Race**				1
Black	8	7 (3.93%)	1 (1.00%)		Black	0	—	—	
Other	5	3 (1.69%)	2 (2.00%)		Other	0	—	—	
White	265	168 (94.4%)	97 (97.0%)		White	170	85 (100%)	85 (100%)	
**Years of diagnosis**				0.921	**Years of diagnosis**				0.81
2004–2009	124	78 (43.8%)	46 (46.0%)		2004–2009	80	38 (44.7%)	42 (49.4%)	
2010–2015	124	81 (45.5%)	43 (43.0%)		2010–2015	72	38 (44.7%)	34 (40.0%)	
2016	30	19 (10.7%)	11 (11.0%)		2016	18	9 (10.6%)	9 (10.6%)	
**Specific stage**				0.452					
IA	198	130 (73.0%)	68 (68.0%)						
IB/IIA	80	48 (27.0%)	32 (32.0%)						
**T**				0.452	**T**				0.213
T1	198	130 (73.0%)	68 (68.0%)		*T*1	128	68 (80.0%)	60 (70.6%)	
T2	80	48 (27.0%)	32 (32.0%)		*T*2	42	17 (20.0%)	25 (29.4%)	
**Surgery type**				0.079	**Surgery type**				0.747
Sublobar resection	93	52 (29.2%)	41 (41.0%)		Sublobar resection	59	31 (36.5%)	28 (32.9%)	
Lobectomy or extended	182	123 (69.1%)	59 (59.0%)		Lobectomy or extended	111	54 (63.5%)	57 (67.1%)	
Pneumonectomy or extended	3	3 (1.69%)	0 (0.00%)		Pneumonectomy or extended	0			
**Tumor size**	278	1.95 [1.40; 2.60]	2.00 [1.50; 2.60]	0.453	**Tumor size**	170	1.90 [1.40; 2.50]	2.00 [1.50; 2.60]	0.463

**Table 2:** Results of univariate Cox proportion hazard analysis for OS and CSS before PSM.

Characteristics	OS		CSS	
	HR [95% CI]	*P* value	HR [95% CI]	*P* value
*Radiation sequence*				
No PORT	Reference			
PORT	0.84 [0.58–1.23]	0.376	0.73 [0.46–1.14]	0.166
*Age*	1.03 [1.01–1.06]	0.005^*∗∗*^	1.02 [1–1.05]	0.084

*Race*				
Black	Reference			
White	1.09 [0.35–3.44]	0.878	0.78 [0.25–2.46]	0.669
Other	1.99 [0.4–9.87]	0.402	1.87 [0.38–9.3]	0.443

*Gender*				
Female	Reference			
Male	1.55 [1.09–2.23]	0.016^*∗*^	1.47 [0.97–2.23]	0.071

*T*				
T1	Reference			
T2	1.07 [0.73–1.58]	0.728	1.17 [0.75–1.83]	0.497

*Surgery type*				
Sublobar resection	Reference			
Lobectomy or extended	0.38 [0.26–0.55]	<0.001^*∗∗∗*^	0.34 [0.22–0.52]	<0.001^*∗∗∗*^
Pneumonectomy or extended	0.22 [0.03–1.6]	0.135	0.3 [0.04–2.22]	0.241

^*∗*^ *P* value < 0.05; ^*∗∗*^*P* value < 0.01; ^*∗∗∗*^*P* value < 0.001.

## Data Availability

Raw data of SCLC patients could be acquired at the SEER database (https://seer.cancer.gov/).

## Abbreviations

SCLC: Small cell lung cancer
TNM: Tumor-node-metastasis
SEER: Surveillance, Epidemiology, And End Results
OS: Overall survival
CSS: Cancer-specific survival
AJCC: American Joint Committee on Cancer
PSM: Propensity score matching
CI: Confidence intervals
HR: Hazard ratio
POCT: Postoperative chemotherapy.

## Supplementary Materials

Supplementary Materials Figure S1: Spearman correlation analysis of extracted variables. Table S1: the raw information of 278 SCLC patients with stage T1-2N0M0. Table S2: variance inflation factors of extracted variables.
