# Supplementary material for: Additional Postoperative Radiotherapy Prolonged the Survival of Patients with I-IIA Small Cell Lung Cancer: Analysis of the SEER Database
Source: J Oncol. 2022 Jun 18;2022:6280538. doi: 10.1155/2022/6280538 (PMC9233591; doi:10.1155/2022/6280538)
Supplement: Supplementary Materials — Figure S1: Spearman correlation analysis of extracted variables. Table S1: the raw information of 278 SCLC patients with stage T1-2N0M0. Table S2: variance inflation factors of extracted variables. [file 6280538.f1.pdf]

Supplementary Material

Figure S1 Spearman correlation analysis of extracted variables.

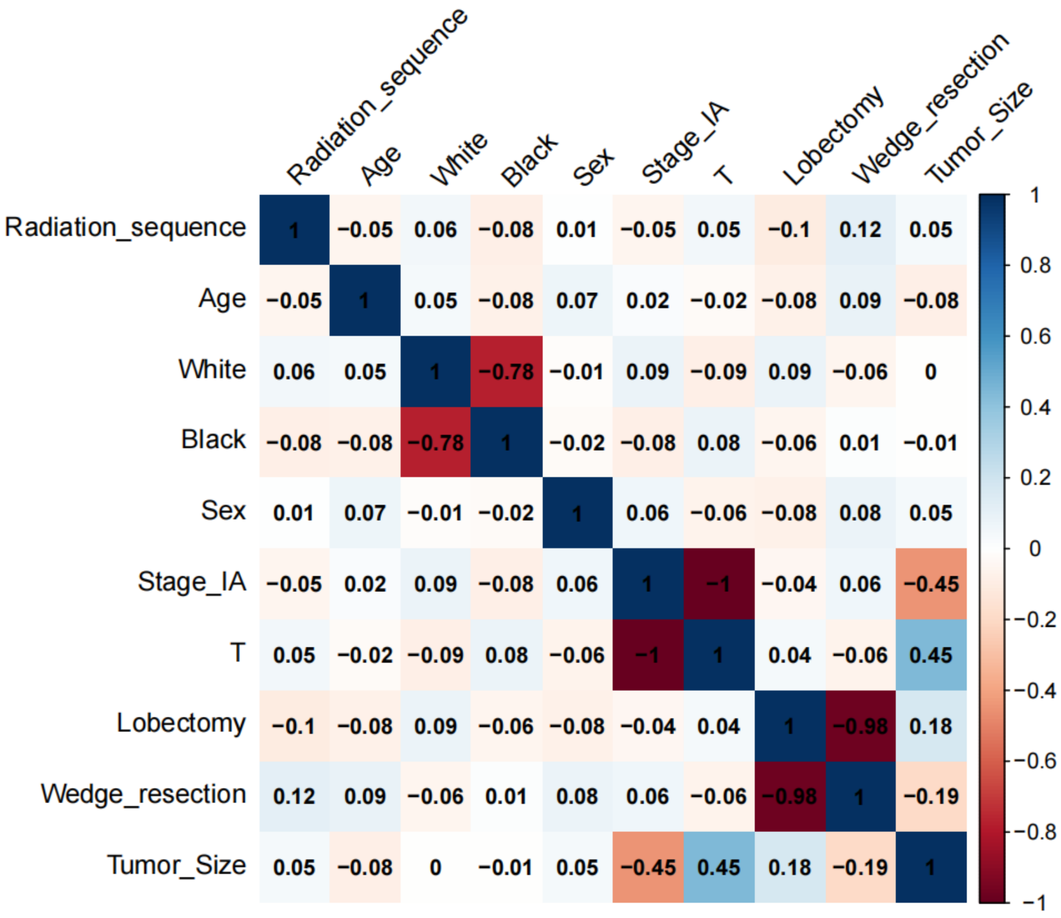

Table S1 The raw information of 278 SCLC patients with stage T1-2N0M0

Patient ID	Radiation sequence	Chemotherapy	Age	Race	Sex	Year of diagnosis	Stage	Specific stage	T	Specific T	N	M	Surgery type	Tumor size	Survival time	OS status	CSS status
999308	1	1	70	White	0	2010-2015	1	IA	1	T1a	N0	M0	II	1.5	82	0	0
4033724	0	1	74	White	0	2010-2015	1	IA	1	T1b	N0	M0	I	3	14	1	1
4121736	1	1	59	White	0	2010-2015	1	IA	1	T1a	N0	M0	II	1.7	36	0	0
4628494	0	1	65	White	0	2010-2015	1	IA	1	T1b	N0	M0	II	3	12	0	0
8428458	0	1	64	White	0	2004-2009	1	IA	1	T1	N0	M0	II	2	31	1	1
8451592	0	1	63	White	0	2004-2009	1	IA	1	T1	N0	M0	II	1.5	38	1	0
8465848	1	1	65	White	0	2004-2009	1	IA	1	T1	N0	M0	II	1.5	112	0	0
8518571	0	1	65	White	1	2004-2009	1	IA	1	T1	N0	M0	II	0.7	96	0	0
8974700	0	1	71	White	0	2010-2015	1	IA	1	T1a	N0	M0	I	1.6	23	1	1
9459091	0	1	62	White	0	2010-2015	1	IA	1	T1a	N0	M0	II	2	45	0	0
9513207	0	1	67	White	0	2010-2015	1	IA	1	T1a	N0	M0	II	2	23	1	1
9536737	0	1	80	White	0	2010-2015	1	IA	1	T1a	N0	M0	II	1.7	12	0	0
9570947	1	1	58	White	1	2016	1	IA	1	p1A	p0	c0	I	1	6	0	0
9573547	0	1	57	White	0	2016	1	IA	1	p1B	p0	c0	II	2.8	9	0	0
13956074	1	1	70	White	1	2004-2009	1	IA	1	T1	N0	M0	I	1	13	1	1
13974202	1	1	62	White	1	2004-2009	1	IA	1	T1	N0	M0	II	2.4	121	1	0
13975646	0	1	67	White	1	2004-2009	1	IA	1	T1	N0	M0	II	3	10	1	1
13990920	0	1	56	White	0	2004-2009	1	IA	1	T1	N0	M0	II	2.5	5	1	1
14497253	0	1	63	White	1	2010-2015	1	IA	1	T1a	N0	M0	I	1.8	38	1	1
14597630	1	1	63	White	1	2010-2015	1	IA	1	T1b	N0	M0	II	2.6	49	0	0
14611553	0	1	56	White	1	2010-2015	1	IA	1	T1a	N0	M0	I	1.4	14	1	1
14623170	0	1	67	White	0	2010-2015	1	IA	1	T1b	N0	M0	II	2.8	38	0	0
15067291	0	1	53	White	0	2010-2015	1	IA	1	T1a	N0	M0	I	0.3	24	0	0
15067567	0	1	67	White	1	2010-2015	1	IA	1	T1a	N0	M0	II	1.6	16	1	1
15097318	0	1	80	White	1	2010-2015	1	IA	1	T1a	N0	M0	II	1.5	15	0	0
15111663	0	1	77	White	1	2010-2015	1	IA	1	T1b	N0	M0	II	2.5	14	0	0
15120355	0	1	81	Black	0	2016	1	IA	1	p1A	p0	c0	II	1.5	7	0	0
15126719	1	1	75	White	0	2016	1	IA	1	p1A	p0	c0	II	1.4	9	0	0
15142359	0	1	57	White	0	2016	1	IA	1	p1B	p0	c0	II	3	4	0	0

15143663	0	1	72	White	1	2016	1	IA	1	p1B	p0	c0	II	2.5	1	0	0
19658644	0	1	61	White	0	2004-2009	1	IA	1	T1	N0	M0	II	2.6	113	0	0
20127694	0	1	63	White	0	2010-2015	1	IA	1	T1b	N0	M0	II	2.2	82	0	0
20170810	1	1	59	White	1	2010-2015	1	IA	1	T1b	N0	M0	II	2.6	17	1	1
22109012	1	1	65	White	0	2004-2009	1	IA	1	T1	N0	M0	II	2.5	100	0	0
22116484	0	1	73	White	0	2004-2009	1	IA	1	T1	N0	M0	II	2.5	91	0	0
22130997	0	1	68	White	0	2004-2009	1	IA	1	T1	N0	M0	II	0.9	89	0	0
22457271	0	1	59	White	1	2010-2015	1	IA	1	T1a	N0	M0	II	1.9	35	1	1
22499296	0	1	68	White	0	2010-2015	1	IA	1	T1b	N0	M0	II	3	57	0	0
22514680	0	1	62	White	1	2010-2015	1	IA	1	T1b	N0	M0	I	2.8	45	0	0
25775108	1	1	66	White	0	2004-2009	1	IA	1	T1	N0	M0	II	2.4	136	1	1
25776656	1	1	70	White	0	2004-2009	1	IA	1	T1	N0	M0	II	2	91	1	0
25834846	0	1	61	White	1	2004-2009	1	IA	1	T1	N0	M0	II	2.3	134	0	0
26318831	0	1	58	White	1	2004-2009	1	IA	1	T1	N0	M0	I	1.2	3	1	1
26323399	0	1	72	White	0	2004-2009	1	IA	1	T1	N0	M0	II	1.5	110	0	0
26424253	1	1	68	White	0	2004-2009	1	IA	1	T1	N0	M0	II	1.5	85	0	0
27462045	0	1	68	White	1	2010-2015	1	IA	1	T1a	N0	M0	I	1.9	13	1	1
27481496	1	1	58	White	1	2010-2015	1	IA	1	T1b	N0	M0	II	2.6	17	0	0
27517902	0	1	58	White	1	2016	1	IA	1	p1A	p0	c0	II	1.8	9	0	0
28964998	0	1	67	White	1	2004-2009	1	IA	1	T1	N0	M0	II	2.6	23	1	1
29444538	0	1	55	White	0	2010-2015	1	IA	1	T1a	N0	M0	II	1.2	13	0	0
29447263	0	1	58	White	1	2016	1	IA	1	p1A	p0	c0	II	1.1	9	0	0
29457768	0	1	70	White	0	2016	1	IA	1	p1A	p0	c0	II	1.9	1	0	0
30980852	0	1	53	White	0	2004-2009	1	IA	1	T1	N0	M0	II	2.8	22	1	0
31384720	0	1	79	White	0	2004-2009	1	IA	1	T1	N0	M0	I	1.1	17	1	1
31432628	0	1	53	White	1	2004-2009	1	IA	1	T1	N0	M0	II	2	77	1	1
31434536	0	1	67	White	1	2004-2009	1	IA	1	T1	N0	M0	II	1.3	16	1	1
31913762	0	1	65	White	0	2010-2015	1	IA	1	T1a	N0	M0	I	1.2	40	0	0
33867994	0	1	66	White	0	2010-2015	1	IA	1	T1b	N0	M0	II	2.1	37	1	1
33979180	0	1	75	White	1	2016	1	IA	1	p1B	p0	c0	I	3	2	0	0
37814737	0	1	53	Black	1	2004-2009	1	IA	1	T1	N0	M0	I	1.5	42	1	1
38246959	0	1	57	Other	1	2004-2009	1	IA	1	T1	N0	M0	II	2	29	1	1
38257061	0	1	80	White	1	2004-2009	1	IA	1	T1	N0	M0	II	1.5	31	1	1

38292608	0	1	77	White	1	2004-2009	1	IA	1	T1	N0	M0	I	1.5	5	1	1
38323338	0	1	56	White	0	2004-2009	1	IA	1	T1	N0	M0	I	0.7	131	0	0
38859931	0	1	57	White	1	2004-2009	1	IA	1	T1	N0	M0	I	1.2	11	1	1
38877745	0	1	78	White	1	2004-2009	1	IA	1	T1	N0	M0	I	1.5	21	1	0
39422498	0	1	74	White	1	2010-2015	1	IA	1	T1a	N0	M0	II	2	26	1	0
41000229	0	1	79	White	1	2016	1	IA	1	p1B	p0	c0	I	2.7	7	0	0
41528180	0	1	57	White	0	2004-2009	1	IA	1	T1	N0	M0	II	1.2	95	0	0
49441475	0	1	72	White	0	2004-2009	1	IA	1	T1	N0	M0	I	2.2	93	1	0
49927329	1	1	71	White	0	2004-2009	1	IA	1	T1	N0	M0	I	2.6	103	1	1
50431885	0	1	74	White	1	2004-2009	1	IA	1	T1	N0	M0	I	0.8	22	1	1
50440005	0	1	48	White	0	2004-2009	1	IA	1	T1	N0	M0	I	0.9	112	1	1
50463902	0	1	64	White	0	2004-2009	1	IA	1	T1	N0	M0	II	1.7	14	1	1
50475070	0	1	58	White	0	2004-2009	1	IA	1	T1	N0	M0	II	1.5	51	1	1
50520148	0	1	83	White	1	2004-2009	1	IA	1	T1	N0	M0	I	1	22	1	1
50960403	0	1	67	White	0	2004-2009	1	IA	1	T1	N0	M0	II	0.9	112	0	0
50977962	0	1	75	White	0	2004-2009	1	IA	1	T1	N0	M0	I	1.2	111	0	0
51014968	1	1	69	White	1	2004-2009	1	IA	1	T1	N0	M0	I	0.9	75	1	0
51628742	0	1	57	White	1	2004-2009	1	IA	1	T1	N0	M0	II	2.6	32	1	1
52069740	1	1	39	White	1	2004-2009	1	IA	1	T1	N0	M0	I	2.9	80	0	0
52134995	0	1	71	Other	0	2004-2009	1	IA	1	T1	N0	M0	I	1.2	28	1	1
53731135	1	1	60	White	0	2010-2015	1	IA	1	T1a	N0	M0	II	2	50	0	0
54269926	1	1	66	White	1	2010-2015	1	IA	1	T1b	N0	M0	II	3	44	0	0
54275791	0	1	76	White	0	2010-2015	1	IA	1	T1a	N0	M0	I	1.4	31	1	1
54347068	0	1	78	White	0	2010-2015	1	IA	1	T1b	N0	M0	I	2.6	22	1	1
54802843	1	1	56	White	0	2010-2015	1	IA	1	T1b	N0	M0	II	2.5	32	0	0
54810510	1	1	67	White	0	2010-2015	1	IA	1	T1a	N0	M0	I	1	30	0	0
54933402	0	1	64	White	1	2010-2015	1	IA	1	T1b	N0	M0	I	2.2	25	0	0
55388040	0	1	67	Other	1	2010-2015	1	IA	1	T1b	N0	M0	II	2.5	17	0	0
55515631	1	1	57	White	0	2016	1	IA	1	p1A	p0	c0	II	1.4	7	0	0
57971685	0	1	58	White	0	2004-2009	1	IA	1	T1	N0	M0	I	1.4	31	1	1
57984928	1	1	60	White	0	2004-2009	1	IA	1	T1	N0	M0	I	2	17	1	1
58001278	1	1	69	White	1	2004-2009	1	IA	1	T1	N0	M0	II	2.5	12	1	1
58022899	1	1	72	White	0	2004-2009	1	IA	1	T1	N0	M0	I	2	18	1	1

58332194	1	1	55	White	1	2004-2009	1	IA	1	T1	N0	M0	I	1	30	1	1
58349700	1	1	64	White	1	2004-2009	1	IA	1	T1	N0	M0	II	1.5	127	0	0
58402768	1	1	65	White	0	2004-2009	1	IA	1	T1	N0	M0	II	1	117	0	0
58424107	0	1	58	White	0	2004-2009	1	IA	1	T1	N0	M0	II	1	31	1	1
58439979	0	1	67	White	1	2004-2009	1	IA	1	T1	N0	M0	II	0.5	105	0	0
58443147	0	1	76	White	0	2004-2009	1	IA	1	T1	N0	M0	II	2.5	70	1	0
58463128	1	1	63	White	1	2004-2009	1	IA	1	T1	N0	M0	I	2	17	1	1
58468880	1	1	57	White	0	2004-2009	1	IA	1	T1	N0	M0	II	1.6	100	0	0
58791772	1	1	60	White	0	2004-2009	1	IA	1	T1	N0	M0	II	1.3	109	0	0
58866559	1	1	52	White	1	2010-2015	1	IA	1	T1a	N0	M0	I	1.2	77	0	0
58904160	1	1	71	White	0	2010-2015	1	IA	1	T1a	N0	M0	I	1	14	1	1
58914989	0	1	57	White	0	2010-2015	1	IA	1	T1a	N0	M0	II	1.2	61	1	1
58917629	0	1	67	White	0	2010-2015	1	IA	1	T1b	N0	M0	I	2.5	10	1	1
59228475	0	1	63	White	0	2010-2015	1	IA	1	T1a	N0	M0	II	1.5	45	1	1
59255430	0	1	70	White	0	2010-2015	1	IA	1	T1a	N0	M0	II	1.5	46	1	0
59256158	0	1	65	White	0	2010-2015	1	IA	1	T1a	N0	M0	I	1	48	0	0
59264867	1	1	64	White	1	2010-2015	1	IA	1	T1a	N0	M0	II	2	42	1	0
59283023	1	1	78	White	0	2010-2015	1	IA	1	T1a	N0	M0	II	1.3	44	0	0
59294075	1	1	59	White	0	2010-2015	1	IA	1	T1b	N0	M0	I	3	28	1	1
59316663	0	1	65	White	1	2010-2015	1	IA	1	T1b	N0	M0	II	2.3	37	0	0
59331344	1	1	50	White	1	2010-2015	1	IA	1	T1b	N0	M0	I	2.9	32	0	0
59342019	0	1	75	White	1	2010-2015	1	IA	1	T1a	N0	M0	II	1.8	33	0	0
59673192	1	1	68	White	0	2010-2015	1	IA	1	T1a	N0	M0	II	1.7	16	0	0
59675796	0	1	68	White	0	2010-2015	1	IA	1	T1a	N0	M0	II	1.7	20	0	0
59706259	0	1	66	White	1	2010-2015	1	IA	1	T1a	N0	M0	II	1	30	0	0
59729342	1	1	67	White	0	2016	1	IA	1	p1B	p0	c0	II	2.2	5	0	0
59756360	1	1	55	White	1	2016	1	IA	1	p1A	p0	c0	I	2	0	0	0
59806555	1	1	61	White	1	2016	1	IA	1	p1B	p0	c0	II	3	2	0	0
60766833	0	1	75	White	0	2004-2009	1	IA	1	T1	N0	M0	I	1	111	0	0
61523517	0	1	63	White	0	2004-2009	1	IA	1	T1	N0	M0	II	1.5	142	0	0
62081670	0	1	49	White	0	2004-2009	1	IA	1	T1	N0	M0	II	2	1	1	0
62112213	1	1	68	White	0	2004-2009	1	IA	1	T1	N0	M0	II	1.9	94	0	0
62640904	1	1	67	White	1	2010-2015	1	IA	1	T1b	N0	M0	I	2.1	8	1	0

62655858	1	1	53	White	1	2010-2015	1	IA	1	T1a	N0	M0	II	1.5	69	0	0
62670435	0	1	59	White	1	2010-2015	1	IA	1	T1b	N0	M0	II	2.2	65	0	0
63274809	0	1	72	White	1	2010-2015	1	IA	1	T1a	N0	M0	II	1.7	33	0	0
63276183	0	1	71	White	1	2010-2015	1	IA	1	T1b	N0	M0	II	3	30	0	0
63307297	1	1	66	Black	1	2010-2015	1	IA	1	T1a	N0	M0	II	0.8	14	1	1
63346880	0	1	52	Black	0	2016	1	IA	1	p1A	p0	c0	II	1.6	10	0	0
63410398	0	1	63	White	0	2016	1	IA	1	p1A	c0	c0	I	1.5	1	0	0
63411295	0	1	68	White	0	2016	1	IA	1	p1A	p0	c0	II	2	7	0	0
70556800	1	1	69	White	0	2004-2009	1	IA	1	T1	N0	M0	II	1.3	147	0	0
70592926	1	1	57	White	1	2004-2009	1	IA	1	T1	N0	M0	I	1.1	84	1	0
70987955	0	1	67	White	1	2004-2009	1	IA	1	T1	N0	M0	II	1.2	155	0	0
71013752	0	1	70	White	0	2004-2009	1	IA	1	T1	N0	M0	II	2.5	140	0	0
71042829	0	1	69	White	1	2004-2009	1	IA	1	T1	N0	M0	I	2	23	1	1
71073015	1	1	78	White	0	2004-2009	1	IA	1	T1	N0	M0	II	1	98	0	0
71090930	0	1	64	White	0	2004-2009	1	IA	1	T1	N0	M0	II	1.3	124	0	0
71554729	0	1	60	White	1	2004-2009	1	IA	1	T1	N0	M0	II	2.5	9	1	1
71616883	0	1	74	White	0	2004-2009	1	IA	1	T1	N0	M0	II	1.8	103	0	0
71624512	0	1	77	White	0	2004-2009	1	IA	1	T1	N0	M0	I	2	24	1	0
71629160	0	1	62	White	0	2004-2009	1	IA	1	T1	N0	M0	II	1.3	26	1	0
71660845	0	1	79	White	0	2004-2009	1	IA	1	T1	N0	M0	II	1.2	19	1	1
71676276	1	1	67	White	1	2004-2009	1	IA	1	T1	N0	M0	I	1.5	97	0	0
72103485	1	1	67	White	1	2004-2009	1	IA	1	T1	N0	M0	I	2.1	30	1	1
72112820	0	1	67	White	0	2004-2009	1	IA	1	T1	N0	M0	II	0.8	90	0	0
72141724	0	1	69	White	1	2004-2009	1	IA	1	T1	N0	M0	II	2.2	84	1	0
72663920	1	1	59	White	1	2010-2015	1	IA	1	T1b	N0	M0	II	2.9	10	1	0
72758181	1	1	65	White	0	2010-2015	1	IA	1	T1a	N0	M0	I	1.1	8	1	0
73171607	0	1	77	White	0	2010-2015	1	IA	1	T1b	N0	M0	II	2.5	51	0	0
73179455	0	1	80	White	0	2010-2015	1	IA	1	T1a	N0	M0	I	1.5	6	1	0
73186474	0	1	65	White	0	2010-2015	1	IA	1	T1a	N0	M0	II	1.4	46	0	0
73188455	1	1	55	White	0	2010-2015	1	IA	1	T1a	N0	M0	I	1.2	45	0	0
73192301	0	1	69	White	0	2010-2015	1	IA	1	T1a	N0	M0	II	1.3	52	0	0
73201433	0	1	62	White	1	2010-2015	1	IA	1	T1a	N0	M0	II	1.5	43	0	0
73285902	0	1	66	White	0	2010-2015	1	IA	1	T1b	N0	M0	I	2.8	15	1	1

73712212	1	1	73	White	0	2010-2015	1	IA	1	T1b	N0	M0	I	2.5	32	0	0
73716475	0	1	72	White	0	2010-2015	1	IA	1	T1a	N0	M0	II	1	25	0	0
73740620	1	1	78	White	1	2010-2015	1	IA	1	T1a	N0	M0	I	1.5	27	0	0
73778957	1	1	68	White	0	2010-2015	1	IA	1	T1a	N0	M0	II	2	21	0	0
73877870	1	1	62	White	0	2016	1	IA	1	p1A	c0	c0	I	1.3	2	0	0
74264158	0	1	67	White	0	2016	1	IA	1	p1A	c0	c0	II	1.7	1	0	0
74353306	0	1	71	White	1	2016	1	IA	1	p1A	c0	c0	I	1.6	7	0	0
75452316	0	1	81	White	0	2004-2009	1	IA	1	T1	N0	M0	I	2.9	116	1	0
75463470	0	1	48	White	0	2004-2009	1	IA	1	T1	N0	M0	II	2.9	23	1	1
75947510	0	1	60	White	0	2004-2009	1	IA	1	T1	N0	M0	I	1	97	1	1
75959693	1	1	61	White	0	2004-2009	1	IA	1	T1	N0	M0	II	1.5	116	0	0
75996955	1	1	65	White	1	2004-2009	1	IA	1	T1	N0	M0	II	2.4	107	0	0
76046112	1	1	61	White	0	2004-2009	1	IA	1	T1	N0	M0	II	1.5	35	1	1
76046874	1	1	66	White	1	2004-2009	1	IA	1	T1	N0	M0	II	2.5	96	0	0
76050395	0	1	63	White	1	2004-2009	1	IA	1	T1	N0	M0	II	2.2	96	0	0
76082544	0	1	68	White	1	2004-2009	1	IA	1	T1	N0	M0	II	2.7	91	0	0
76083791	1	1	72	White	1	2004-2009	1	IA	1	T1	N0	M0	II	2.1	50	1	0
76500669	0	1	66	White	1	2004-2009	1	IA	1	T1	N0	M0	I	2	87	0	0
76567304	1	1	78	White	1	2010-2015	1	IA	1	T1a	N0	M0	II	1.8	42	1	1
76567316	0	1	51	White	1	2010-2015	1	IA	1	T1b	N0	M0	II	2.2	79	0	0
76568382	1	1	75	White	0	2010-2015	1	IA	1	T1a	N0	M0	I	1.3	41	1	1
76572916	0	1	62	White	0	2010-2015	1	IA	1	T1a	N0	M0	II	0.9	61	1	0
76590854	0	1	69	White	1	2010-2015	1	IA	1	T1b	N0	M0	II	2.1	20	1	1
76595605	0	1	71	White	1	2010-2015	1	IA	1	T1a	N0	M0	III	1.1	58	1	1
76607970	1	1	74	White	0	2010-2015	1	IA	1	T1b	N0	M0	II	2.1	64	0	0
76660376	0	1	69	White	1	2010-2015	1	IA	1	T1a	N0	M0	II	1	55	0	0
77114107	0	1	59	White	1	2010-2015	1	IA	1	T1a	N0	M0	II	1.1	5	0	0
77125703	1	1	82	White	0	2010-2015	1	IA	1	T1b	N0	M0	I	3	43	0	0
77129133	0	1	69	White	1	2010-2015	1	IA	1	T1b	N0	M0	II	2.2	34	1	1
77140666	0	1	68	White	1	2010-2015	1	IA	1	T1b	N0	M0	II	2.5	36	0	0
77214710	0	1	69	White	1	2010-2015	1	IA	1	T1a	N0	M0	II	1.5	2	1	0
77220153	1	1	65	White	1	2010-2015	1	IA	1	T1a	N0	M0	I	1.4	26	0	0
77225300	0	1	73	White	1	2010-2015	1	IA	1	T1a	N0	M0	I	1.3	16	0	0

77230690	0	1	59	White	0	2010-2015	1	IA	1	T1a	N0	M0	I	1.5	18	0	0
77700282	1	1	85	White	1	2016	1	IA	1	p1A	p0	c0	I	1.4	6	0	0
77715200	0	1	68	White	1	2016	1	IA	1	p1A	p0	c0	II	1.5	6	0	0
77723348	0	1	52	White	0	2016	1	IA	1	p1B	p0	c0	I	2.9	0	0	0
3409253	1	1	65	Other	1	2004-2009	1	IB/IIA	2	T2	N0	M0	I	2.6	90	1	1
3504827	0	1	79	White	1	2004-2009	1	IB/IIA	2	T2	N0	M0	II	1.6	42	1	1
3966886	0	1	79	White	1	2004-2009	1	IB/IIA	2	T2	N0	M0	II	2.5	18	1	1
4086706	0	1	49	White	0	2010-2015	1	IB/IIA	2	T2a	N0	M0	II	3.2	62	1	1
4588937	0	1	79	White	1	2010-2015	1	IB/IIA	2	T2a	N0	M0	II	3.1	17	1	1
8487378	0	1	53	White	0	2004-2009	1	IB/IIA	2	T2	N0	M0	II	1.3	115	0	0
9500264	0	1	66	White	1	2010-2015	1	IB/IIA	2	T2a	N0	M0	II	3	7	0	0
9504760	1	1	63	White	0	2010-2015	1	IB/IIA	2	T2a	N0	M0	II	3.2	24	1	1
13486901	0	1	51	Black	0	2010-2015	1	IB/IIA	2	T2a	N0	M0	I	1.8	20	1	1
13487611	0	1	62	White	0	2004-2009	1	IB/IIA	2	T2	N0	M0	II	1.8	11	1	1
14035392	1	1	67	White	1	2004-2009	1	IB/IIA	2	T2	N0	M0	II	4.4	97	1	0
14057826	0	1	79	Black	0	2004-2009	1	IB/IIA	2	T2	N0	M0	II	3.8	89	0	0
14078625	1	1	52	White	0	2004-2009	1	IB/IIA	2	T2	N0	M0	II	4.8	85	0	0
14490634	1	1	70	White	0	2010-2015	1	IB/IIA	2	T2a	N0	M0	II	1.5	79	0	0
14509088	0	1	80	White	1	2010-2015	1	IB/IIA	2	T2a	N0	M0	II	2.1	76	0	0
15040601	0	1	67	White	1	2010-2015	1	IB/IIA	2	T2a	N0	M0	I	2.3	24	1	1
15132523	1	1	56	White	1	2016	1	IB/IIA	2	p2A	c0	c0	I	1.5	6	0	0
19649755	1	1	51	White	0	2004-2009	1	IB/IIA	2	T2	N0	M0	II	2.3	119	0	0
20080550	0	1	64	White	1	2004-2009	1	IB/IIA	2	T2	N0	M0	I	3.6	86	1	1
20121606	0	1	57	White	1	2010-2015	1	IB/IIA	2	T2a	N0	M0	I	1.8	79	0	0
20680657	0	1	58	White	0	2016	1	IB/IIA	2	p2A	p0	c0	II	4	10	0	0
22085413	0	1	79	White	0	2004-2009	1	IB/IIA	2	T2	N0	M0	II	3.5	17	1	1
22098618	1	1	76	White	0	2004-2009	1	IB/IIA	2	T2	N0	M0	I	1.1	25	1	1
22503967	0	1	57	Black	1	2010-2015	1	IB/IIA	2	T2a	N0	M0	I	2.3	45	0	0
22515799	0	1	60	White	0	2010-2015	1	IB/IIA	2	T2a	N0	M0	II	4.5	38	0	0
26402092	1	1	64	White	0	2004-2009	1	IB/IIA	2	T2	N0	M0	II	4.2	6	1	1
27461285	1	1	73	White	1	2010-2015	1	IB/IIA	2	T2a	N0	M0	II	3.1	23	0	0
27496801	1	1	67	White	0	2010-2015	1	IB/IIA	2	T2a	N0	M0	II	2.3	13	0	0
31383501	1	1	70	White	1	2004-2009	1	IB/IIA	2	T2	N0	M0	II	4.4	113	0	0

31430357	0	1	73	White	0	2004-2009	1	IB/IIA	2	T2	N0	M0	II	3.5	91	0	0
31479117	0	1	71	White	1	2010-2015	1	IB/IIA	2	T2a	N0	M0	I	2.8	12	1	0
31983493	0	1	73	White	0	2010-2015	1	IB/IIA	2	T2a	N0	M0	II	1.3	17	0	0
40568495	1	1	74	Other	0	2010-2015	1	IB/IIA	2	T2a	N0	M0	I	2.2	8	0	0
49305385	0	1	77	White	1	2004-2009	1	IB/IIA	2	T2	N0	M0	II	3.4	12	1	1
49321880	0	1	57	White	0	2004-2009	1	IB/IIA	2	T2	N0	M0	II	3.3	108	1	0
49326339	0	1	40	White	1	2004-2009	1	IB/IIA	2	T2	N0	M0	II	4	144	0	0
49386739	1	1	69	White	1	2004-2009	1	IB/IIA	2	T2	N0	M0	I	4.7	9	1	0
52081055	1	1	68	White	0	2004-2009	1	IB/IIA	2	T2	N0	M0	II	3.1	91	0	0
52088498	1	1	65	White	0	2004-2009	1	IB/IIA	2	T2	N0	M0	I	2	3	1	1
52196978	0	1	78	White	0	2010-2015	1	IB/IIA	2	T2a	N0	M0	II	1.3	60	1	0
53158204	0	1	55	White	0	2010-2015	1	IB/IIA	2	T2a	N0	M0	II	4	72	0	0
53224329	1	1	48	White	0	2010-2015	1	IB/IIA	2	T2a	N0	M0	II	3.5	42	0	0
54344825	0	1	74	White	0	2010-2015	1	IB/IIA	2	T2a	N0	M0	II	3.3	37	0	0
55392012	0	1	74	White	1	2010-2015	1	IB/IIA	2	T2a	N0	M0	I	2.5	16	0	0
55933467	0	1	72	White	0	2016	1	IB/IIA	2	p2A	p0	c0	II	4.5	10	0	0
57971085	1	1	59	White	1	2004-2009	1	IB/IIA	2	T2	N0	M0	II	4.5	34	1	1
58330091	0	1	57	White	0	2004-2009	1	IB/IIA	2	T2	N0	M0	II	3.4	139	0	0
58349787	0	1	53	White	0	2004-2009	1	IB/IIA	2	T2	N0	M0	II	3	131	0	0
58367496	1	1	63	White	0	2004-2009	1	IB/IIA	2	T2	N0	M0	I	4.1	31	1	1
58404346	1	1	69	White	0	2004-2009	1	IB/IIA	2	T2	N0	M0	I	2.5	12	1	1
58779894	1	1	67	White	0	2004-2009	1	IB/IIA	2	T2	N0	M0	II	2	94	1	0
58805128	0	1	70	White	0	2004-2009	1	IB/IIA	2	T2	N0	M0	II	1.8	14	1	1
58834082	0	1	43	White	0	2010-2015	1	IB/IIA	2	T2a	N0	M0	III	5	81	0	0
59236218	0	1	61	White	1	2010-2015	1	IB/IIA	2	T2a	N0	M0	II	3.3	14	1	1
59240591	1	1	63	White	0	2010-2015	1	IB/IIA	2	T2a	N0	M0	I	2	29	1	1
59250328	0	1	51	White	0	2010-2015	1	IB/IIA	2	T2a	N0	M0	II	3.2	48	0	0
59304615	1	1	62	White	0	2010-2015	1	IB/IIA	2	T2a	N0	M0	I	3.8	22	1	1
59341916	0	1	61	White	1	2010-2015	1	IB/IIA	2	T2a	N0	M0	II	4.5	32	0	0
59680888	0	1	71	White	1	2010-2015	1	IB/IIA	2	T2a	N0	M0	II	0.6	16	0	0
59729511	1	1	55	White	0	2016	1	IB/IIA	2	p2A	p0	c0	II	3.1	6	0	0
59751346	0	1	62	White	0	2016	1	IB/IIA	2	p2A	p0	c0	II	3.4	2	0	0
60253929	0	1	56	White	0	2004-2009	1	IB/IIA	2	T2	N0	M0	II	2.5	142	0	0

62751973	1	1	55	White	0	2010-2015	1	IB/IIA	2	T2a	N0	M0	II	3.7	13	0	0
63294906	1	1	71	White	0	2010-2015	1	IB/IIA	2	T2a	N0	M0	I	3.5	29	0	0
70526601	1	1	79	White	1	2004-2009	1	IB/IIA	2	T2	N0	M0	I	1.5	13	1	0
71123772	0	1	71	White	0	2004-2009	1	IB/IIA	2	T2	N0	M0	I	1.4	18	1	1
71604084	0	1	78	White	0	2004-2009	1	IB/IIA	2	T2	N0	M0	II	1.7	22	1	1
72098631	0	1	70	White	0	2004-2009	1	IB/IIA	2	T2	N0	M0	I	1.5	11	1	1
72193896	1	1	49	White	0	2010-2015	1	IB/IIA	2	T2a	N0	M0	II	1.5	76	0	0
72655707	1	1	67	White	1	2010-2015	1	IB/IIA	2	T2a	N0	M0	I	1.1	27	1	1
73298645	0	1	80	White	1	2010-2015	1	IB/IIA	2	T2a	N0	M0	I	3.9	22	1	1
73711484	0	1	53	White	0	2010-2015	1	IB/IIA	2	T2a	N0	M0	II	4	23	0	0
73725293	0	1	65	White	1	2010-2015	1	IB/IIA	2	T2a	N0	M0	II	3.7	27	0	0
73782613	1	1	75	White	1	2010-2015	1	IB/IIA	2	T2a	N0	M0	II	4	18	0	0
75528761	0	1	57	Black	0	2004-2009	1	IB/IIA	2	T2	N0	M0	III	3.2	123	0	0
75949088	1	1	74	White	1	2004-2009	1	IB/IIA	2	T2	N0	M0	II	4	90	1	0
76082845	0	1	70	White	1	2004-2009	1	IB/IIA	2	T2	N0	M0	II	2.1	41	1	1
76575056	0	1	66	White	0	2010-2015	1	IB/IIA	2	T2a	N0	M0	II	1.6	73	0	0
77229036	0	1	60	White	0	2010-2015	1	IB/IIA	2	T2a	N0	M0	II	1.6	28	0	0
77717175	1	1	70	White	1	2016	1	IB/IIA	2	p2A	p0	c0	II	2.6	3	0	0

Notes: in surgical type, I:Sublobar resection; II:Lobectomy or extended ; III:Pneumonectomy or extended)

Table S2 Variance inflation factors of extracted variables

	GVIF	Df	GVIF^(1/(2*Df))
dt$Radiation_sequence	1.035157	1	1.017427
dt$Race	1.072959	2	1.017761
dt$Sex	1.027404	1	1.013609
dt$Age	1.032692	1	1.016214
dt$T	1.398647	1	1.182644
dt$Surgery_type	1.127868	2	1.030539
